# Role of Perfusion Index as a Predictor of Vasopressor Requirement in Patients With Sepsis

**DOI:** 10.7759/cureus.100431

**Published:** 2025-12-30

**Authors:** Mirza Nahiduzzaman, Suman Kundu, Manas K Mazumder, MD T Islam, Shubamay D Nath, Rajeebshankar Karmakar, Masnoon Ahmed Noor

**Affiliations:** 1 Critical Care Medicine, National Gastroliver Institute and Hospital, Dhaka, BGD; 2 Critical Care Medicine, Kuwait Bangladesh Friendship General Hospital, Dhaka, BGD; 3 Anesthesia and Critical Care, Bangladesh Medical University, Dhaka, BGD; 4 Critical Care Medicine, Rangpur Medical College, Rangpur, BGD; 5 Critical Care Medicine, Chattagram Maa-O-Shishu Hospital Medical College, Chattagram, BGD; 6 Thoracic Surgery, Faridpur Medical College, Faridpur, BGD; 7 Thoracic Surgery, Bangladesh Medical University, Dhaka, BGD

**Keywords:** iv vasopressor, perfusion index, perfusion index (pi), s: perfusion index, s: sepsis, vasopressor, vasopressor requirements, vasopressor support, vasopressor therapy, vasopressor use

## Abstract

Background

Sepsis remains a leading cause of intensive care unit (ICU) admission and is frequently associated with organ dysfunction and high mortality due to impaired microvascular perfusion. As peripheral hypoperfusion is central to septic shock, reliable bedside tools capable of detecting early microcirculatory failure are critically important. Although mean arterial pressure (MAP) and serum lactate are routinely used in sepsis assessment, both have notable limitations. MAP may remain preserved through compensatory vasoconstriction despite ongoing microcirculatory collapse, while lactate elevation is often delayed and influenced by multiple metabolic factors. In this context, the perfusion index (PI), derived from pulse oximetry, has emerged as a direct and quantitative marker of peripheral perfusion. PI represents the ratio of pulsatile arterial blood flow to non-pulsatile tissue blood flow and reflects arterial inflow and microvascular tone. In septic shock, circulatory and microcirculatory derangements, including vasodilation and flow maldistribution, reduce effective arterial pulsatility, leading to a decline in PI. This physiological basis supports the role of PI as an early, non-invasive predictor of vasopressor requirement in patients with sepsis.

Methods

This prospective observational study was carried out in the ICU of Dhaka Medical College Hospital, enrolling 96 patients with sepsis who met specific inclusion and exclusion criteria. Detailed demographic, clinical, and investigative data were systematically recorded, and both PI and lactate levels were measured before fluid resuscitation. The need for vasopressor support within 24 hours was then assessed and correlated with baseline PI to determine its predictive significance.

Result

The study population was predominantly middle-aged, with a mean age of 48.2 years, and two-thirds were male, reflecting a typical mixed-ICU demographic. Comorbidities were common, particularly diabetes and combined hypertension-diabetes, alongside notable burdens of coronary artery disease and chronic airway disease. Patients were admitted with a wide range of critical illnesses, spanning trauma, respiratory infections such as pneumonia, neurological emergencies, postoperative states, and metabolic derangements. Vital signs on admission showed physiological stress, with elevated heart and respiratory rates and a broad range of arterial pressures, nearly half presenting with MAP <65 mmHg. When comparing vasopressor with non-vasopressor groups, heart rate (p=0.001) and MAP (p<0.001) showed significant differences, identifying them as strong markers of hemodynamic decline. PI values were split between low and high groups, but most hypotensive patients clustered within the low-PI category, illustrating a clear physiological relationship. The PI ≤0.3 threshold identified nearly all patients with MAP <65 mmHg, reinforcing its validity as an early perfusion marker. Diagnostic performance metrics further demonstrated PI’s strength, achieving a sensitivity of 91.3%, specificity of 94%, and high predictive values in both directions.

Conclusion

PI serves as a reliable early predictor of vasopressor requirement in patients with sepsis before fluid resuscitation.

## Introduction

Sepsis is a life-threatening condition resulting from a dysregulated immune response to infection, often leading to multiorgan dysfunction involving the lungs, kidneys, liver, heart, and brain [1]. The pathophysiology involves a complex interplay of inflammation, coagulation, and circulatory failure that causes tissue hypoperfusion and cellular injury [2]. Endothelial disruption results in vasodilation, capillary leakage, and edema, which can progress to acute respiratory distress syndrome, acute kidney injury, hepatic cholestasis, gastrointestinal translocation, and septic encephalopathy [1]. Despite advances in management, sepsis continues to carry a high global mortality rate [3]. Early hemodynamic optimization, including fluid resuscitation and vasopressor therapy, is crucial for improving outcomes, as timely intervention has been shown to reduce mortality [4].

Current sepsis management focuses on restoring systemic and microcirculatory perfusion through maintaining mean arterial pressure (MAP) using vasopressors such as norepinephrine [5-7], although optimal timing and individualized pressure targets remain debated [8]. Monitoring tissue perfusion is a cornerstone of septic shock management, as persistent hypoperfusion despite resuscitation predicts poor outcomes [9]. Peripheral perfusion serves as an early and sensitive marker of circulatory failure [10]. The perfusion index (PI) is defined as the ratio of the pulsatile (AC) component of the pulse oximetry signal, generated by arterial inflow with each cardiac cycle, to the non-pulsatile (DC) component arising from venous blood, capillaries, bone, and surrounding tissues, expressed as a percentage [4]. PI directly reflects peripheral arterial pulsatility and microvascular tone, with higher values indicating improved perfusion [10,11]. In septic shock, profound circulatory and microcirculatory abnormalities, including vasodilation and flow maldistribution, reduce effective arterial pulsatility, leading to a decline in PI. As early vasopressor initiation has been associated with improved survival, this study aimed to evaluate the role of PI as a predictor of vasopressor requirement in patients with sepsis [4,5].

## Materials and methods

Study design

**Figure 1:**
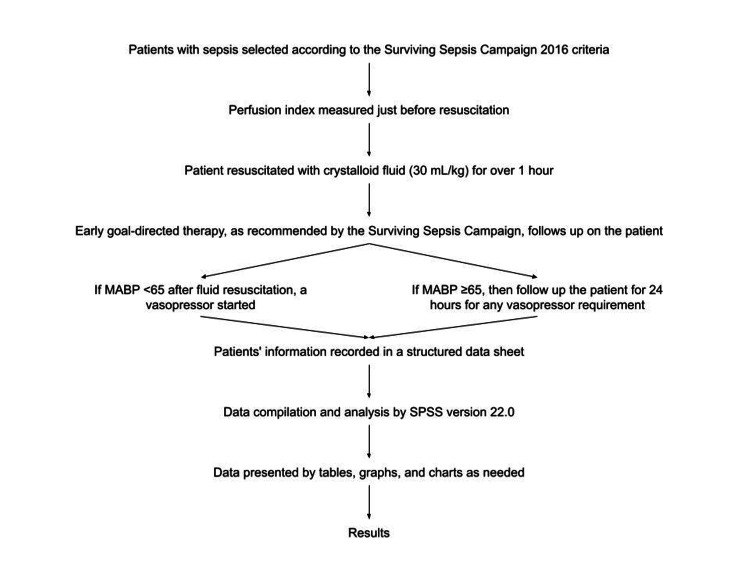
Study flow chart MABP, mean arterial blood pressure.

A total of 96 male and female patients with sepsis who met the inclusion and exclusion criteria were enrolled after obtaining informed written consent. Each participant was clearly informed about the study objectives, potential benefits, and possible risks. Demographic and clinical information were recorded in detail. Before resuscitation, PI was measured. The patients were then managed following the early goal-directed therapy protocol recommended by the Surviving Sepsis Campaign [12]. Vital signs, including heart rate, MAP, temperature, and respiratory rate, were carefully recorded, along with baseline laboratory investigations and arterial blood gas analysis. The PI and SpO₂ were measured using a portable pulse oximeter (model JX1130BL, HealthTree, China), applying the probe to the index finger after signal stabilization. Under aseptic precautions, 6 mL of blood was drawn from each patient before resuscitation for routine laboratory tests. Patients were closely followed for hemodynamic changes and vital parameters. If any developed shock or failed to maintain a MAP ≥65 mmHg after receiving 30 mL/kg of intravenous fluids, vasopressor therapy was initiated. Vasopressor use was defined as the requirement of any vasopressor agent within 24 hours of intensive care unit (ICU) admission after initial resuscitation. The Sepsis-related Organ Failure Assessment (SOFA) score was used to quantify disease severity and organ dysfunction. Data collection was performed using a pre-structured data sheet, with meticulous checking to identify and correct potential errors (Figure 1).

Data recording

All demographic and clinical details of the study participants were recorded in individual case record forms. The PI values were obtained directly from the pulse oximeter readings.

Statistical analysis

Collected data were carefully checked, edited, and encoded before being entered into SPSS version 22.0 (IBM Corp., Armonk, NY) and Microsoft Excel (Microsoft Corp., Redmond, WA, USA) for statistical analysis. The Kolmogorov-Smirnov test was used to assess the normality of data distribution. Quantitative variables were expressed as mean ± standard deviation and compared using the Student’s t-test for two groups. Qualitative or categorical variables were presented as frequencies and proportions, with comparisons made using the chi-square test when appropriate. Graphical and tabular presentations, including pie charts, bar diagrams, histograms, and curves, were used to visualize findings. The receiver operating characteristic (ROC) curve was constructed to assess the predictive value of PI for vasopressor requirement, and the area under the ROC curve was calculated to evaluate the discriminative ability of these markers. All analyses were performed at a 5% level of significance (p<0.05).

Ethical considerations

Ethical clearance for the study was obtained from the Academic and Institutional Review Board (IRB) of the Bangladesh Medical University, Shahbagh, Dhaka, and necessary permissions were also secured from the relevant departments of the institute. In adherence to the Helsinki Declaration of 2011 concerning medical research involving human subjects, all participants were thoroughly informed about the study design and objectives before their inclusion. They were explicitly assured of their right to withdraw from the study at any point and for any reason, without any consequence. Written informed consent was obtained from each participant through a transparent and respectful process that clearly outlined the potential benefits and risks associated with the procedure. Confidentiality was strictly maintained, and no data were disclosed without the explicit permission of the respondents. No force was applied, and interviews were conducted only with those who willingly agreed to participate.

## Results

As shown in Table 1, the study population was predominantly middle-aged with a modest skew toward male participants, reflecting the typical demographic pattern observed in many mixed-ICU cohorts. Table 2 illustrates a substantial burden of chronic comorbidities, with metabolic, cardiovascular, and respiratory diseases featuring prominently, highlighting a population already predisposed to hemodynamic instability and complex ICU trajectories. The diagnostic spectrum in Table 3 paints an even broader clinical landscape, encompassing trauma, severe respiratory infections, neurological insults, postoperative states, metabolic crises, and other acute medical conditions, demonstrating the heterogeneous yet clinically realistic mix of cases.

**Table 1:** Distribution of the study subjects considering age and sex SD, standard deviation.

Variables	Number (n)	Percentage (%)
Age (years)		
18-30	13	13.5
31-40	27	28.1
41-50	35	36.4
51-60	14	14.6
60-65	7	7.3
Mean ± SD	48.2 ± 8.1	
Sex		
Male	65	67.7
Female	31	32.3

**Table 2:** Distribution of the patients according to comorbidity CAD, coronary artery disease; COPD, chronic obstructive pulmonary disease; DM, diabetes mellitus; HTN, hypertension.

Comorbidity	Number (n)	Percentage (%)
HTN	14	14.6
Diabetes	26	27.1
HTN and DM	27	28.1
CAD	20	20.8
COPD/bronchial asthma	21	21.9

**Table 3:** Distribution of the patients according to diagnosis in ICU BA, bronchial asthma; COPD, chronic obstructive pulmonary disease; ICU, intensive care unit; PPE, postpartum eclampsia.

Diagnosis in ICU	Number (n)	Percentage (%)
Head injury and polytrauma	10	10.4
Respiratory disease		
Pneumonia	9	9.4
Acute exacerbation of COPD/BA	5	5.2
Neurological disease		
Stroke	6	6.2
Meningoencephalitis	5	5.2
Seizure disorder	2	2.1
Neurosurgical status	5	5.2
Post-surgical status	12	12.5
PPE, eclampsia	5	5.2
Others (infectious disease)	8	8.3
Metabolic encephalopathy	6	6.2
Poisoning	4	4.2
Liver failure	3	3.1
Acute pancreatitis	2	2.1

As reflected in Table 4, the study cohort exhibited clear signs of physiological stress at baseline, with elevated cardiorespiratory parameters and broadly variable arterial pressures suggesting early hemodynamic compromise. Table 5 further reveals that nearly half of the patients already presented with hypotension, framing a population in which perfusion failure and vasopressor need were expected clinical concerns. The contrast becomes more pronounced in Table 6, where patients who eventually required vasopressors showed markedly higher heart and respiratory rates and substantially lower MAPs, with both heart rate and MAP demonstrating significant differences (p=0.001 and p<0.001, respectively), highlighting their strong alignment with circulatory deterioration. Table 7 paints a similarly compelling picture through the lens of peripheral perfusion, where almost half of the cohort had conspicuously reduced PI values, signaling impaired microvascular flow even before overt shock physiology emerged. This pattern is reinforced in Table 8, where low PI values corresponded overwhelmingly to hypotensive states, highlighting PI’s strong validity as an early marker of vasopressor requirement.

**Table 4:** Distribution of study patients according to vital signs and hemodynamic parameters SD, standard deviation.

Variables	Mean ± SD	Range
Temperature (°C)	37.93 ± 1.18	36.7-38.9
Heart rate (bpm)	102.19 ± 19.24	83-120
Respiratory rate (breaths/min)	28.02 ± 5.91	22-34
Mean arterial pressure (mmHg)	74.75 ± 23.43	50-98

**Table 5:** Distribution of study patients according to mean arterial pressure (mmHg) findings

Mean arterial pressure (mmHg)	Number of patients (n)	Percentage of patients (%)
<65	46	47.9
≥65	50	52.1

**Table 6:** Comparison of vital signs between patients requiring and not requiring vasopressors

Vital signs	Vasopressor (n=45)	No vasopressor (n=51)	p-value
Temperature (ºC)	37.25 ± 1.12	38.62± 1.26	0.584
Heart rate (bpm)	116.23 ± 22.4	88.15 ± 15.9	0.001
Respiratory rate (breaths/min)	32.19 ± 7.15	23.86 ± 4.62	0.085
Mean arterial pressure (mmHg)	62.94 ± 18.09	86.52 ± 29.65	<0.001

**Table 7:** Results of the perfusion index among the study patients SD, standard deviation.

Perfusion index	Number of patients, n (%)	Mean ± SD
≤0.3	45 (46.8)	0.16 ± 0.08
>0.3	51 (53.1)	1.54 ± 0.25

**Table 8:** Evaluation of the validity of perfusion index as a predictor of vasopressor requirement

Perfusion index	Mean arterial pressure (mmHg)	
	<65 mmHg, n (%)	≥65 mmHg, n (%)
≤0.3	42 (43.7)	3 (3.12)
>0.3	4 (4.17)	47 (48.96)

**Figure 2:**
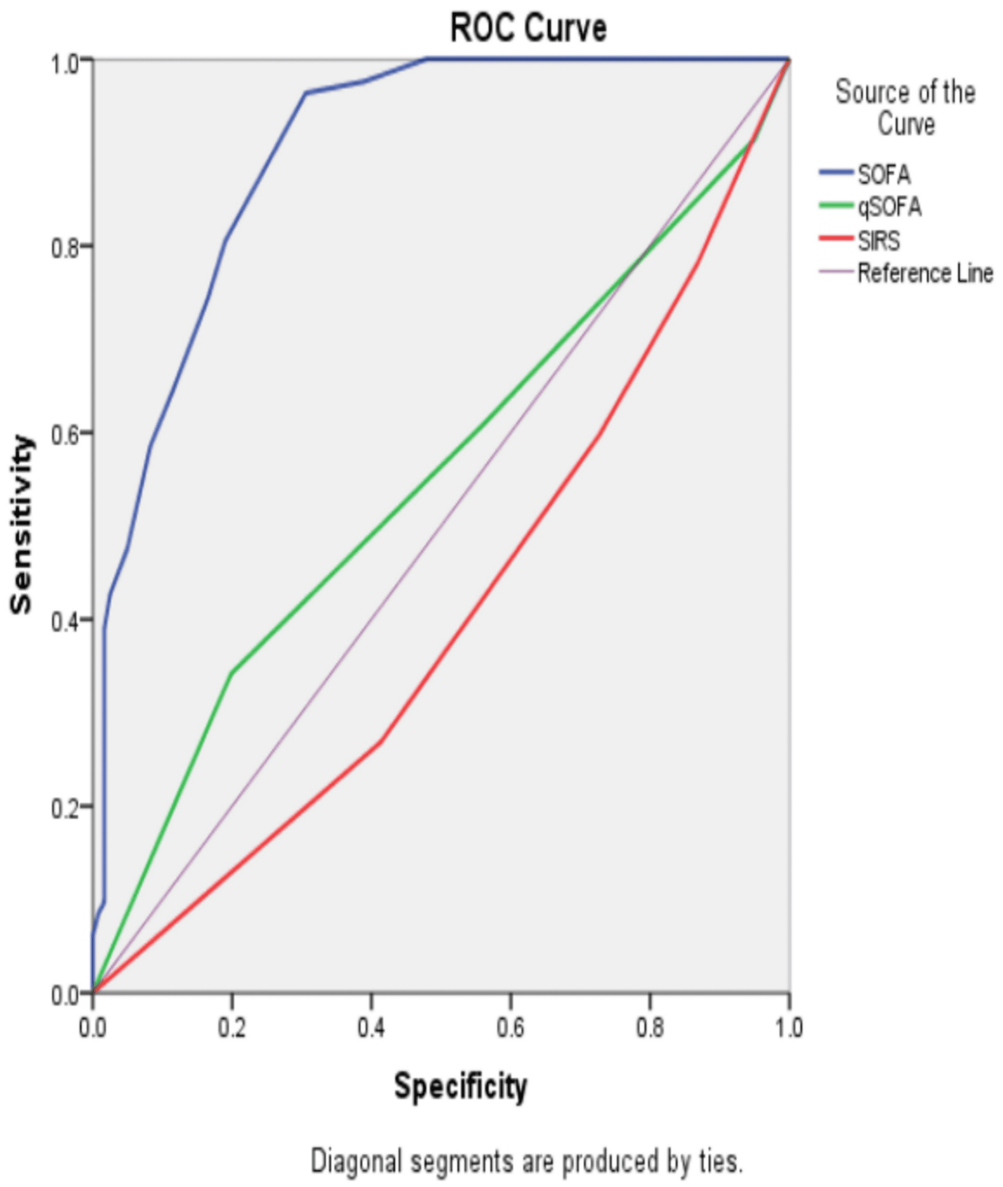
ROC curve of PI value in prediction of vasopressor requirement PI, perfusion index; qSOFA, quick Sepsis-related Organ Failure Assessment; ROC, receiver operating characteristic; SIRS, systemic inflammatory response syndrome; SOFA, Sepsis-related Organ Failure Assessment.

In Table 9, PI demonstrated a highly reliable diagnostic profile for predicting vasopressor requirement, with predominance of correct classifications and only minimal misidentification. This translated into excellent sensitivity, specificity, and predictive values, collectively indicating that PI captures early circulatory compromise with remarkable precision in patients with sepsis. The accompanying Figure 2 further reinforces this performance, where the ROC curve illustrates that PI’s discriminatory ability is superior to traditional scoring systems, showing a trajectory that rises sharply toward the upper left corner, reflecting strong sensitivity at progressively higher specificity thresholds. The graphical separation in Figure 2 clearly supports the robustness of PI as a diagnostic tool.

**Table 9:** Diagnostic performance of perfusion index in predicting vasopressor requirement in patients with sepsis

Parameter	Value
True positive (TP)	42 (43.75%)
False positive (FP)	3 (3.13%)
False negative (FN)	4 (4.17%)
True negative (TN)	47 (48.95%)
Sensitivity	91.3%
Specificity	94.0%
Positive predictive value (PPV)	93.3%
Negative predictive value (NPV)	92.1%

## Discussion

Sepsis is a major cause of illness and death in ICUs, and finding simple, non-invasive ways to predict the need for vasopressors early can greatly improve patient outcomes. This study explored the prognostic value of the PI in predicting vasopressor requirement in patients with sepsis, alongside the evaluation of demographic and clinical correlates. The age distribution in our cohort reflects a predominantly middle-aged population, with most patients clustering in the fourth and fifth decades of life and a smaller representation from both younger and older age groups. This pattern differs meaningfully from several published reports: one study shows a mean age of 51 ± 19 years, closely aligning with our general age profile, while another study reports a mean of 50 ± 17.9 years, which is also comparable [4,13]. A separate investigation notes a markedly older median age of 70 years [14]. Regarding sex distribution, our study demonstrated a modest predominance of male patients, a trend frequently observed in many ICU populations. This finding is consistent with one study showing 69.6% male participants, whereas another study reports a nearly equal distribution with 50% male representation [15,16]. One report shows a higher proportion of female patients, with 62% women among the study sample, highlighting the diversity of demographic patterns across different critical care environments [13]. Our cohort showed a diverse distribution of comorbid conditions, with metabolic, cardiovascular, and respiratory illnesses appearing prominently, an expected pattern in critically ill patients with sepsis. Hypertension and diabetes were among the most frequent chronic conditions, often occurring individually or in combination, while coronary artery disease and chronic airway diseases also contributed substantially to the underlying clinical burden. When compared with published literature, interesting contrasts emerge. One study shows hypertension rates of 51.2%, and another reports 53%, both markedly higher than what we observed [14,17]. For diabetes, our prevalence aligns with one study showing 35.8%, but differs from another reporting only 18%, while a third study notes 29%, suggesting wide variability across populations [14,17,18]. Regarding coronary artery disease, our dataset reflects a moderate proportion, striking a balance between one study showing 36.6% and another reporting a much lower 9% [14,17]. The prevalence of chronic obstructive pulmonary disease or bronchial asthma in our sample closely mirrors that reported in one study (22%) and is slightly higher than the prevalences (19% and 16%) reported in other studies [14,17,18]. Our cohort reflected a wide clinical spectrum typical of a mixed medical-surgical ICU, with patients presenting across trauma, respiratory, neurological, postoperative, infectious, and metabolic categories. Respiratory illnesses, neurological events, and postoperative cases formed substantial portions of the case mix, while metabolic derangements, liver dysfunction, and poisoning appeared less frequently, contributing to a clinically diverse but balanced distribution. When compared with international data, striking contrasts become evident. For instance, while pneumonia appeared in only a modest proportion of our population, one study shows it accounted for 49% of ICU sepsis cases, another reports 53%, and a third documents 38%, highlighting a far heavier respiratory disease burden elsewhere [14,17,18]. One study notes only 5.6%, demonstrating significant epidemiologic variability [4]. Meningoencephalitis constituted a modest share in our cohort, aligning with one study showing 1.3% and another reporting 2.8% [17,18]. Stroke contributed a smaller fraction of admissions in our study, comparable to one study showing 2.8% [19]. Post-surgical patients represented a meaningful subgroup in our ICU, paralleling findings of 16.7% reported in another cohort [18]. Liver failure appeared far less frequently in our sample, whereas one study shows rates as high as 38% and another reports 12% [14,18]. 

The baseline vital signs reflected a pattern of physiological stress typical of early sepsis, with patients presenting with modest pyrexia, elevated cardiorespiratory parameters, and a wide range of arterial pressures that suggested varying degrees of hemodynamic compromise. When compared with findings from other studies, several meaningful parallels and contrasts emerge. For temperature, one study shows a median of 37.1°C, closely approximating our overall trend, whereas another reports a higher median of 38.5°C, reflecting more pronounced febrile responses in some populations [4,15]. Heart rate values in our cohort, though elevated, were slightly lower than those described in one study showing a median of 120 bpm, yet similar to another reporting 104 bpm, indicating that tachycardia remains a consistent physiological signature across septic cohorts [4,15]. Respiratory rates in our cohort aligned closely with one report showing 27 breaths/min, but they were lower than another documenting 35 breaths/min, suggesting variability in the severity of respiratory distress at presentation [4,14]. MAP in our sample demonstrated a broad distribution, sitting between findings from one study showing a median of 63 mmHg and another reporting 70 mmHg, both consistent with the hemodynamic instability typical of early sepsis [4,15]. The distribution of patients above and below the hemodynamic threshold for hypotension revealed a nearly even split, reflecting a population in which early circulatory instability was common but not universal. This balance contrasts meaningfully with patterns described elsewhere. One study shows an almost identical distribution of hypotensive (50%) and normotensive (50%) patients at admission, closely mirroring the overall trend observed in our population [4]. Another study reports a markedly different pattern, with only 25% of patients presenting with MAP below 65 mmHg and 75% maintaining levels above this threshold, suggesting a generally more hemodynamically stable cohort [4]. Patients who required vasopressor support showed a clear pattern of physiological deterioration, with more pronounced tachycardia, higher respiratory drive, and substantially lower arterial pressures, while temperature differences did not reach statistical significance (p=0.584). The contrasts in heart rate and MAP were particularly striking, both demonstrating significant associations with vasopressor use (p=0.001 and p<0.001, respectively), highlighting their strong linkage to evolving circulatory failure. When viewed against international findings, these trends align well with previously published data. One study shows median heart rates of around 120 bpm in shock patients compared with lower rates in more stable individuals, while another reports a baseline median of 104 bpm in similar populations, reflecting tachycardia as a consistent marker of severity [4,15]. Respiratory rates, although elevated in our vasopressor group, echo the heightened medians reported in one study showing 35 breaths/min among critically ill patients with sepsis [4]. The profound drop in arterial pressure observed in our vasopressor group parallels findings of a median 63 mmHg in unstable patients, compared with higher values such as 70 mmHg observed in more stable cohorts [4,15].

The distribution of PI values revealed two nearly balanced groups, with a substantial proportion of patients demonstrating markedly reduced peripheral perfusion. When examined in relation to arterial pressure, those with low PI values overwhelmingly clustered within the hypotensive group, while higher values corresponded strongly with preserved blood pressure, illustrating a coherent and clinically intuitive relationship between PI and hemodynamic stability. The alignment between low PI and hypotension is striking and reinforces its diagnostic relevance. When compared with published literature, one study shows a far greater predominance of patients falling into the low PI category, with 61.1% having values ≤0.3 and only 38.9% above that threshold, suggesting that the severity of microcirculatory failure at presentation may vary significantly across settings [4]. The same study reports the identical 61.1% vs. 38.9% distribution when PI is analyzed in relation to MAP, further emphasizing the strong association between peripheral perfusion deficits and hypotension.

In our study, the PI demonstrated an excellent diagnostic profile for identifying patients who required vasopressor support, with a high proportion of correct classifications and only minimal misidentification, resulting in strong sensitivity, specificity, and predictive values. When compared with external literature, the performance aligns closely with international observations. One research shows a sensitivity of 100% and specificity of 93%, values that mirror the high accuracy observed in our cohort [4]. While our sensitivity is slightly lower and specificity slightly higher than those reported by others, the overall diagnostic strength remains consistent.

## Conclusions

PI emerged in this study as a promising early indicator for identifying patients with sepsis who are likely to require vasopressor support, reflecting its ability to capture subtle changes in microcirculatory perfusion. By providing a simple, non-invasive window into tissue perfusion, PI holds potential to guide clinicians in anticipating vasopressor needs and tailoring resuscitation strategies more effectively. These findings should be interpreted within the context of certain limitations. The study was conducted in a single tertiary care ICU with a modest sample size, which may limit its generalizability to broader settings. Although the focus on early resuscitation yielded meaningful clinical insights, the absence of long-term outcome measures, such as mortality, organ recovery, or persistent organ dysfunction, prevents a full appraisal of PI’s prognostic value across the entire trajectory of sepsis. These strengths and limitations suggest that while PI may serve as a valuable adjunct in early sepsis management, further multicenter studies with extended follow-up are needed to confirm and expand upon its clinical utility.

## Figures and Tables

**Figure 3:**
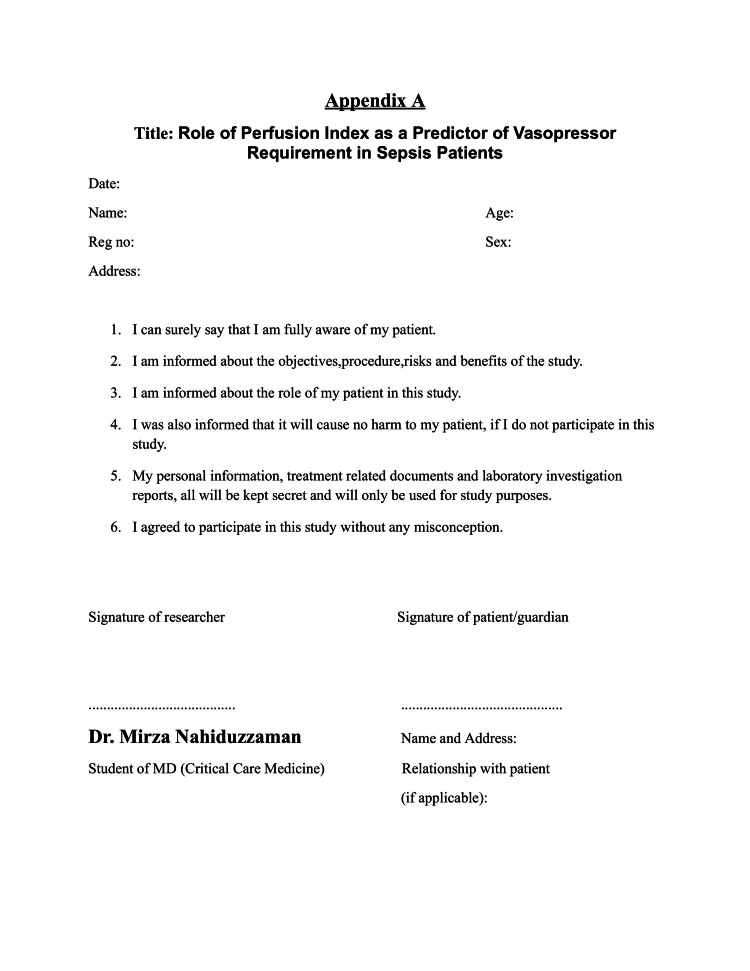
Consent form (English)

**Figure 4:**
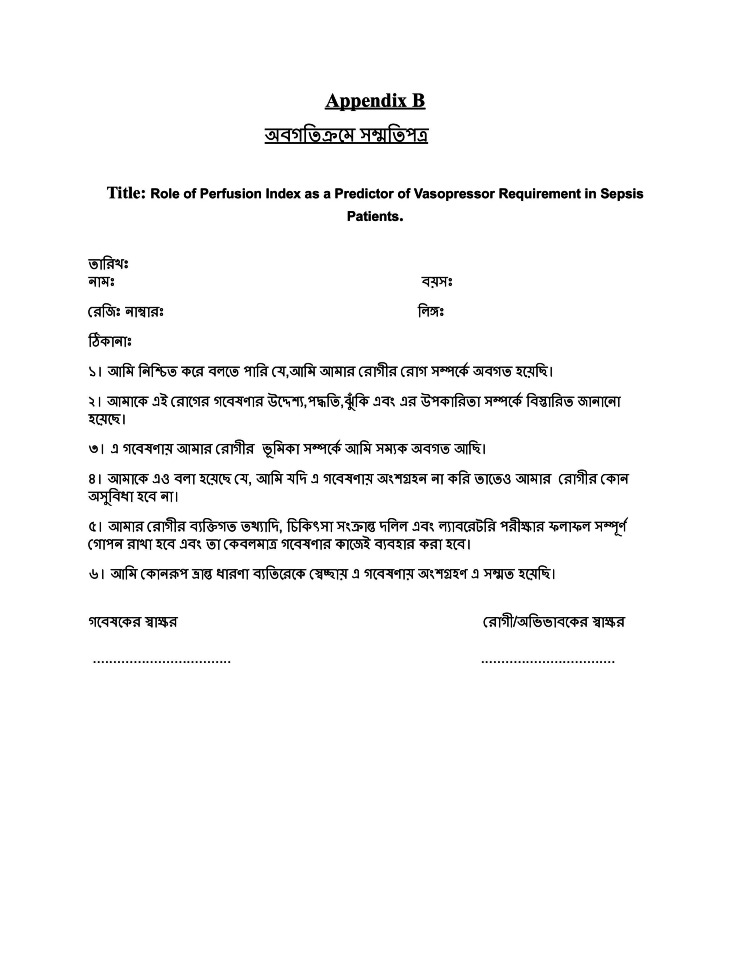
Consent form (Bangla)

**Figure 5:**
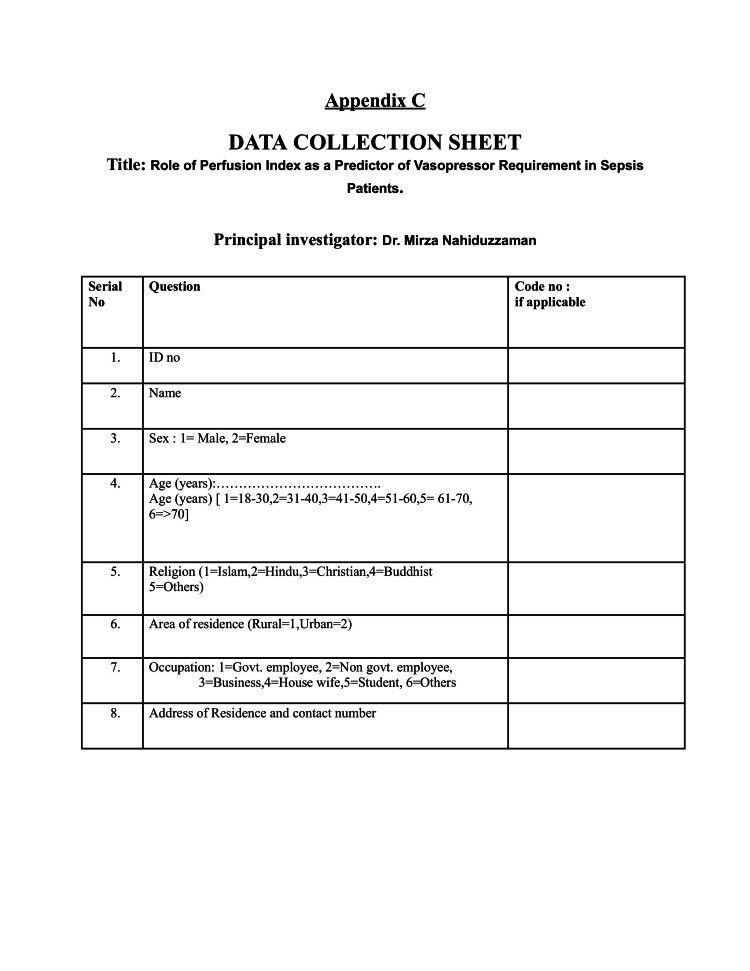
Data collection form 1

**Figure 6:**
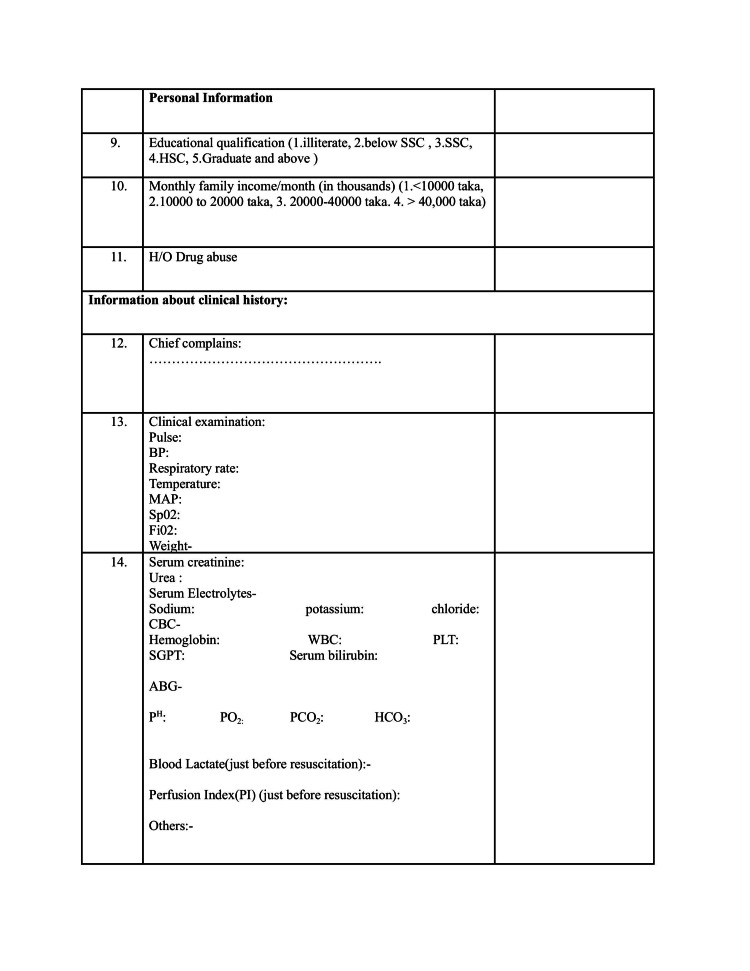
Data collection form 2

**Figure 7:**
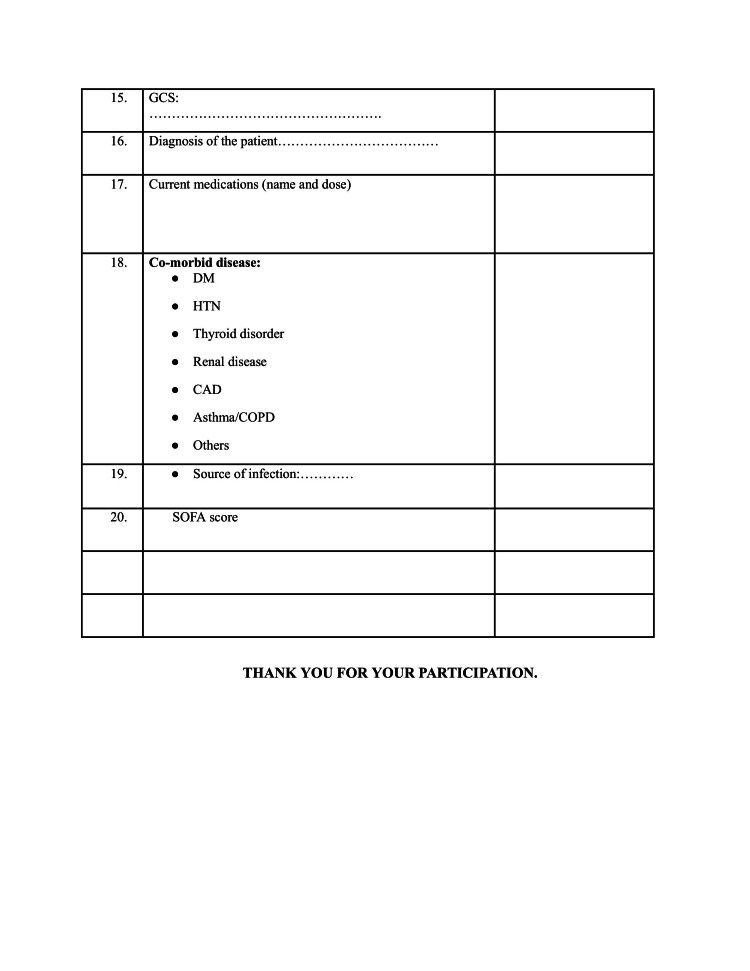
Data collection form 3
